# MicroRNA Networks Modulate Oxidative Stress in Cancer

**DOI:** 10.3390/ijms20184497

**Published:** 2019-09-11

**Authors:** Yang-Hsiang Lin

**Affiliations:** Liver Research Center, Chang Gung Memorial Hospital, Linkou, Taoyuan 333, Taiwan; yhlin0621@cgmh.org.tw; Tel.: +886-3-3281200 (ext. 7785)

**Keywords:** oxidative stress, MicroRNA, signal transduction, therapeutic target

## Abstract

Imbalanced regulation of reactive oxygen species (ROS) and antioxidant factors in cells is known as “oxidative stress (OS)”. OS regulates key cellular physiological responses through signal transduction, transcription factors and noncoding RNAs (ncRNAs). Increasing evidence indicates that continued OS can cause chronic inflammation, which in turn contributes to cardiovascular and neurological diseases and cancer development. MicroRNAs (miRNAs) are small ncRNAs that produce functional 18-25-nucleotide RNA molecules that play critical roles in the regulation of target gene expression by binding to complementary regions of the mRNA and regulating mRNA degradation or inhibiting translation. Furthermore, miRNAs function as either tumor suppressors or oncogenes in cancer. Dysregulated miRNAs reportedly modulate cancer hallmarks such as metastasis, angiogenesis, apoptosis and tumor growth. Notably, miRNAs are involved in ROS production or ROS-mediated function. Accordingly, investigating the interaction between ROS and miRNAs has become an important endeavor that is expected to aid in the development of effective treatment/prevention strategies for cancer. This review provides a summary of the essential properties and functional roles of known miRNAs associated with OS in cancers.

## 1. Introduction

**Figure 1:**
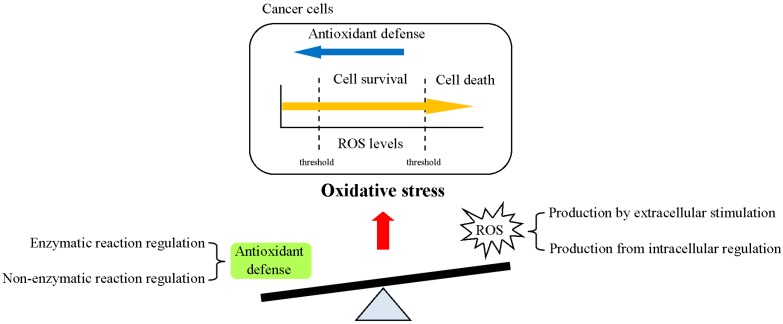
Reactive oxygen species (ROS) production and antioxidant defense in the control of redox homeostasis in cancer cells. Disruption of redox homeostasis by ROS (intra- or extracellular signals) and antioxidant defense (enzymatic or non-enzymatic reactions) induces oxidative stress (OS) and results in various cell functions. The physiological function of ROS is dependent on its concentration. Elevated ROS production and accumulation lead to cell apoptosis. On the other hand, medium levels of ROS promote cell survival and progression.

Imbalanced regulation of reactive oxygen species (ROS) and antioxidant factors in cells is known as “oxidative stress (OS)” (Figure 1). OS drives key cellular physiological regulatory responses through signal transduction, transcription factors (TFs) and noncoding RNAs (ncRNAs) [1]. ROS are oxygen-containing products and are formed during cellular oxidative metabolism. ROS, including superoxide anion (O_2_^−^), hydroxyl radical (OH^−^), hydrogen peroxide (H_2_O_2_), nitric oxide (NO) and singlet oxygen (^1^O_2_), play important roles in cell differentiation, cell death, cell growth, signal transduction, cell apoptosis and chemoresistance [2,3]. Dual roles have been proposed for ROS in biological phenotypes according to their cellular level [4]. High levels of ROS promote cell apoptosis, while low levels of ROS act as a signal transducer to induce cell survival (Figure 1). Recently, excessive ROS production was identified in several cancers where they were significantly correlated with tumorigenesis. However, the underlying mechanism of ROS regulation in cancer development remains unclear.

**Figure 2:**
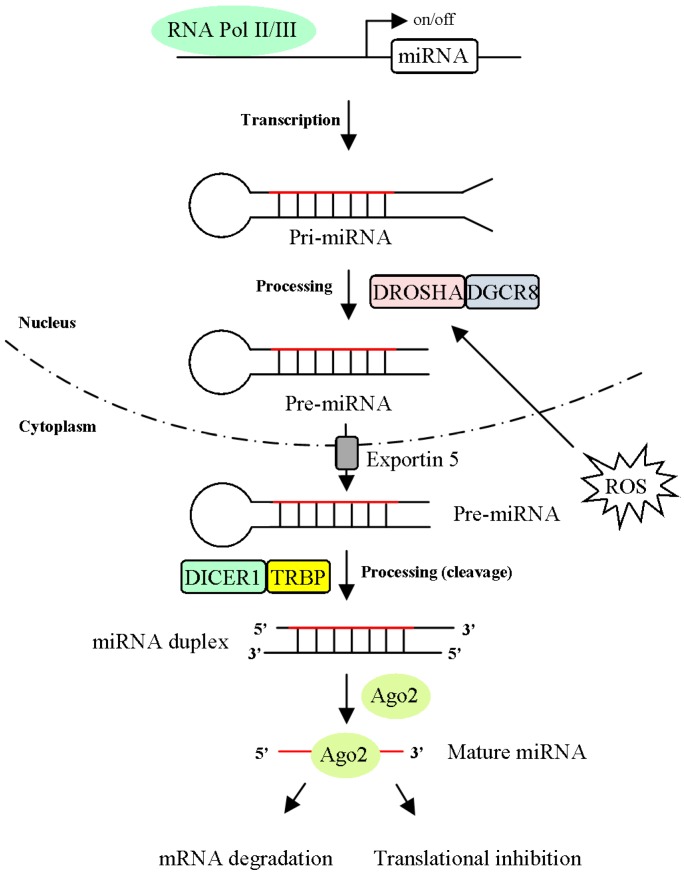
The biogenesis and regulation mechanisms of microRNAs (miRNAs). MiRNAs are transcribed by RNA polymerase II/III and generated the primary miRNA transcript (pri-miRNA). The pri-miRNAs are cleaved into precursor miRNA transcript (pre-miRNA) by the microprocessor complex, a combination of DROSHA and DGCR8. Pre-miRNA is exported to cytoplasm via exportin 5 and further processed by the RNase III enzyme Dicer with the cofactor protein TRBP to generate an approximately 18-25-nt duplex. Either 5p or 3p strand of the mature miRNA (red line) interacts with Argonaute (Ago) protein and forms a miRNA-induced silencing complex (miRISC). There are two models (mRNA degradation and translational repression) of miRNA-mediated gene silencing.

MicroRNAs (miRNAs) are small ncRNA comprising 18-25-nucleotide functional RNA molecules that play critical roles in the regulation of target gene expression by binding to complementary regions of mRNA and regulating mRNA degradation or inhibiting translation (Figure 2). Previous studies have demonstrated that miRNAs are significantly associated with tumor growth, metastasis and cancer progression [5,6]. Based on these findings, dysregulated miRNA expression is a hallmark of cancer.

Cross-talk between ROS and miRNAs has been implicated in cancer development, and it is important to identify the nature of this connection. Interestingly, some specific miRNAs, called ROS-miRs or redoximiRs, are regulated by OS and modulate target gene expression in response to ROS [7,8]. Mesenguer et al. [9] demonstrated that the OS/NFκB axis induced miR-9/9* expression and inhibited expression of its target genes, GTPBP3, MTO1 and TRMU, in MELAS cells. On the other hand, a previous study indicated that miR-21 regulated ROS homeostasis and suppressed the antioxidant response in human umbilical vein endothelial cells (HUVECs) [10]. These findings suggest that ROS could be upstream regulators or downstream effectors of miRNAs. In this review, we focus on how ROS affect biological phenotypes through miRNA and how miRNAs regulate ROS-mediated function in cancer.

## 2. Regulation of ROS Homeostasis in Cells

OS promotes both nuclear and mitochondrial DNA damage and initiates DNA repair pathways [11]. Furthermore, cellular ROS levels can be produced by different mechanisms, such as ionizing radiation, UV radiation, inflammatory cells and chemotherapy. ROS are primarily generated in cells through the byproducts of leaked electrons from the mitochondrial electron transport chain (ETC). Mutations or aberrantly expressed nuclear or mitochondrial genes encoding the ETC components can influence the electron transfer reaction that leads to electron leakage. The electrons are captured by O_2_, producing O_2_^−^, which is usually converted to H_2_O_2_ by manganese (Mn)-containing mitochondrial superoxide dismutase (MnSOD or SOD2), Cu/Zn-containing cytosolic SOD1 or SOD3 [12]. Subsequently, H_2_O_2_ can attack chromosomal DNA and subsequently induce DNA damage. On the other hand, O_2_^−^ can be generated through a reaction catalyzed by some enzymes, including the membrane-located NAD(P)H oxidase complex (NOX), which consists of NOX1-4, endoplasmic reticulum-associated xanthine oxidase (XO), cytochrome c oxidase and cyclooxygenase in some cancer cells [13]. In fact, H_2_O_2_ plays an important role in carcinogenesis because it is capable of diffusing throughout the cell components and producing cellular injury. The injurious effects of ROS in mammalian cells are mediated by the hydroxyl radical (·OH). The generation of OH in vivo is produced in the presence of reduced transition metals, including Co, Cu, Fe, or Ni, mainly through the Fenton reaction [14]. Notably, the ·OH-induced DNA damage includes the generation of 8-hydroxyguanosine (8-OHG), in which the hydrolysis product is 8-hydroxydeoxyguanosine (8-OHdG). 8-OHdG is the most widely used marker of radical attack on DNA. Notably, 8-OHdG is strongly correlated with cancer progression, including that of breast cancer, colorectal cancer, ovarian cancer and hepatocellular carcinoma (HCC) [15,16,17]. For example, hepatic 8-OHdG levels are useful biomarkers for identifying hepatitis C virus (HCV) infection in patients [18]. Alternatively, cells maintain ROS homeostasis by reducing ROS production and triggering specific antioxidant mechanisms to neutralize ROS or mitigate OS [19]. In fact, antioxidant enzymes include SODs, catalase, peroxiredoxins (PRDXs), thioredoxins, glutathione peroxidase and heme oxygenase. First, SOD converts O_2_^−^ to O_2_ or H_2_O_2_. Then, catalase and glutathione peroxidase subsequently convert H_2_O_2_ to H_2_O and O_2_.

## 3. MiRNAs and Their Roles in Oxidative Stress

Previous studies indicated that ROS can induce or suppress miRNA expression and contribute to downstream biological function through regulation of target genes [20]. Increasing evidence has shown cross-talk between miRNAs and components of redox signaling [21,22]. The transcription, biogenesis, translocation and function of miRNAs are highly correlated with ROS, and miRNAs may regulate the expression of redox sensors and other ROS modulators, such as the key components of cellular antioxidant machinery. Redox sensors have been identified and they include transcription factors (e.g., p53, NFκB, c-Myc and nuclear factor erythroid 2 related factor 2 (NRF2)) and kinases (e.g., Akt and IKK), which trigger cellular redox signaling. Here, we summarize how miRNAs are regulated by ROS at the posttranscriptional and transcriptional levels and how the miRNA/ROS axis controls tumorigenesis.

### 3.1. MiRNA Processing is Regulated by ROS

Recently, it was reported that miRNAs can be transcribed by RNA polymerase II/III as longer primary transcripts called primary miRNAs (pri-miRNAs). The mature form of miRNA is generated by the two-step processing of pri-miRNA and is subsequently associated with the effector RNA-induced silencing complex (RISC). The biogenesis and function of miRNAs regulated by ROS are described. Two key genes (*Dicer* and *Drosha*) are mediated by the miRNA processing pathway (Figure 2). A report showed that the expression of Dicer was downregulated by aging-related OS in cerebromicrovascular endothelial cells (CMVECs) [23]. Downregulating Dicer dramatically reduced miRNA expression under H_2_O_2_ treatment compared with the expression of the control. Notably, knocking down Dicer suppressed ROS production in human microvascular endothelial cells (HMECs) [24]. These findings indicated that Dicer expression is part of a feedback loop that modulates ROS production and maintains cellular homeostasis. Upon OS, the expression of pre-miRNA and miRNA in myoblasts is decreased through DGCR8/heme oxygenase-1 (HMOX1) regulation [25]. Heme is required for DGCR8 activity, and the heme-binding domain of DGCR8 plays a crucial role in pri-miRNA recognition for miRNA processing by DROSHA.

### 3.2. ROS Regulate miRNA Expression through the Modulation of Transcription Factors

Accumulating studies have investigated the miRNAs regulated by ROS/TFs such as c-myc, p53, c-Jun, HIF and NFκB [20,26]. This section summarizes how miRNAs are regulated by ROS/TF at the transcriptional level.

ROS exposure has been shown to be correlated with oncogenic signals such as those transduced by c-Myc and Ras [27,28]. c-Myc, a well-known oncogene, is involved in tumor growth, migration, invasion, metabolism and metastasis through the regulation of gene expression. c-Myc activation induces DNA damage in normal human fibroblasts. This effect has been correlated with ROS generation. Expression levels of miR-15a/16, miR-23a, miR-29 and miR-34 family members were downregulated by c-Myc [29]. Overexpression of miR-15a/16 suppressed cell proliferation, angiogenesis, migration and invasion through inhibition of FGF2 in vitro and in vivo [30]. Furthermore, hypoxia-induced suppression of miR-15/16 expression was directly regulated by c-Myc. By contrast, miR-17-92 and miR-221/222 expression is stimulated by c-Myc [29]. The expression levels of miR-17-92 were remarkably inhibited by triptolide in a c-Myc-dependent manner, which resulted in the induction of target genes, including PTEN, BIM and p21, in HCC cells [31]. Moreover, this suppressive effect contributed to enhanced triptolide-induced cell apoptosis.

P53, a tumor suppressor gene, regulates the cell cycle, apoptosis, growth and metabolism through modulation of target genes. P53 is involved in regulating the drosha-dedicated pri-miRNA processing pathway [32]. In addition, p53 modulates miRNA transcription, such as miR-17-92, miR-34a and miR-200c. Interestingly, stress-regulated miRNAs, namely, miR-34 and miR-200, are upregulated in a p53-dependent manner [33,34]. MiR-34 has been implicated as a tumor suppressor because it suppresses the epithelial-mesenchymal transition (EMT), which promotes cancer cell metastasis. Its expression level is positively associated with p53. Importantly, p53 suppresses Snail expression by interacting with miR-34. A study indicates that miR-200c is upregulated upon H_2_O_2_ treatment in endothelial cells and that it contributes to cell apoptosis and senescence through inhibition of the target gene ZEB1 [35]. Moreover, knockdown of p53 can reverse H_2_O_2_-induced miR-200c expression [34].

As mentioned above, exposure to ROS induces chronic inflammation. NFκB acts as a master mediator of the inflammatory response to regulate innate and adaptive immune functions. MiRNA (miR-9, miR-21, miR-30b, miR-146a, miR-155 and miR-17-92 cluster) expression was identified and found to be directly transcriptionally regulated by NFκB [36,37]. Bazzoni et al. [38] indicated that miR-9-1 was induced by lipopolysaccharide (LPS) in a MyD88- and NF-κB-dependent manner. DNA damage activated miR-21 expression through recruitment of NF-κB and signal transducer and activator of transcription 3 (STAT3) to its promoter region and contributed to promoting cell invasion in breast cancer [39]. Another study reported that miR-21 expression and function were mediated by ROS in highly metastatic breast cancer cell lines [40]. In addition, miR-21 induced by ROS via NF-κB activity was involved in arsenic-induced cell transformation [41]. NF-κB bound to the promoter region of the miR-17-92 cluster was identified using chromatin immunoprecipitation (ChIP) assay and was further confirmed by luciferase reporter assay [42]. On the other hand, multiple miRNAs have been identified and have been found to modulate NF-κB activity. MiR-126a was shown to target IκBα, an NFκB inhibitor, and promoted the NFκB signaling pathway [43]. MiR-506 inhibited the expression of the NFκB p65 subunit and led to the production of ROS and p53-dependent apoptosis in lung cancer cells [44]. Notably, miR-506 was regulated by p53. These findings indicated that miR-506 was involved in the p53/NFκB signaling pathway.

NRF2 is a member of the Cap’n’Collar (CNC) family of basic leucine zipper (bZIP) transcription factors [45]. Previously, the actin-binding protein kelch-like ECH-associated protein 1 (KEAP1) was identified as a repressor of NRF2 via proteasomal degradation [46]. NRF2 is involved in antioxidant metabolism, protein degradation, inflammation and radioresistance [47]. Notably, miRNAs can be both indirectly and directly regulated by NRF2 [47,48]. Singh et al. [49] group demonstrated that NRF2 repressed miR-1 and miR-206 expression and led to reprogram glucose metabolism in cancer cells. Furthermore, miR-29 and miR-125b were identified as direct target genes of NRF2 [50,51]. Upregulation of miR-125b by NRF2 resulted in the repression of aryl hydrocarbon receptor repressor and protection of cancer cells from drug-induced toxicity [51]. On the other hand, NRF2 gene was regulated by miRNAs such as miR-28, miR-34a, miR-93 and miR-200a [52,53,54,55]. MiR-28 has been shown to interact with NRF2 3’UTR and represses NRF2 expression in breast cancer cells [52]. Overexpression of miR-34a suppressed NRF2 and NRF2 target genes expressions [53]. Functionally, miR-34a was involved in NRF2-dependent antioxidant pathway in liver. These findings suggested that NRF2 and miRNAs formed a regulatory network and regulated cellular functions.

### 3.3. ROS Regulate miRNA Expression via Epigenetic Regulation

Recently, epigenetic modifications/regulations of the genome have been explored and associated with cancer progression [56]. Changes in the structure or conformation at the nuclear or mitochondrial DNA (nDNA and mtDNA) or RNA level, but not the DNA/RNA sequence, are called epigenetic marks. The main epigenetic alterations in humans are DNA methylation and histone modification, which includes methylation, acetylation and phosphorylation. Aberrant miRNA expression in cancers was discovered and found to be controlled by epigenetic regulation. Promoter regions of miR-125b and miR-199a are hypermethylated through DNMT1 during H_2_O_2_ treatment, as determined using methylation-specific PCR and bisulfate sequencing [57]. Moreover, these two miRNAs are downregulated by ROS in ovarian cancer cells. The level of histone acetylation has an important role in activating gene expression through chromatin remodeling. In contrast, the gene is silenced by histone deacetylases (HDACs), which promote the deacetylation of lysine residues. MiR-466h-5p acts in a proapoptotic role by directly targeting antiapoptotic genes such as BCL2L2 [58]. ROS induce miR-466h-5p expression through inhibition of HDAC2 and result in increased apoptosis.

## 4. Interplay between Oxidative Stress, miRNA and Cancer Development

OS has been reported to contribute to neurological disorders, hypertension, diabetes and cancers. This section focuses on the associations of OS and hypoxia, angiogenesis, metastasis, metabolism, cancer stem cell and senescence, which are all involved in cancer progression.

### 4.1. Association between OS, miRNA and Hypoxia

Hypoxia, known as reduced oxygen availability, mostly occurs in the center of tumors due to the high proliferation ability of cancer cells and abnormal vasculature [59]. Hypoxia-inducible factor 1α (HIF1α) is the master regulator in hypoxia. The activation of HIF1α promotes the expression of several genes, including protein-encoding genes and ncRNAs, and facilitates stem cell renewal, cancer cell survival, metabolism and chemoresistance. The HIF1 transcription factor consists of three hypoxia-induced α subunits (HIF1α/2α/3α) and one β subunit (HIF1β). HIF1α is stabilized and activates a downstream signaling pathway mediated by ROS [60]. Some evidence suggests that telomerase activity is associated with ROS in HCC [61]. Moreover, ROS-mediated telomerase activity is dependent on HIF1α [62]. Expression levels of the human telomerase reverse transcriptase gene (hTERT) are upregulated by HIF1α. Specific binding sites for HIF1α in the hTERT promoter regions were identified by luciferase and ChIP assays. In addition, cancer stem cell (CSC) markers, OCT4 and Notch, are induced by HIF1α and promote stem cell renewal. Expression levels of SOX2 and KLF4 are positively regulated by ROS in glioblastoma cells [63]. HIF1α and ROS activation are responsible for regulating glucose transporter 1 (GLUT1), hexokinase II (HKII) and glutaminase expression and the reprogramming of cancer cell metabolism [64]. Moreover, miR-210 acts in an oncogenic role in cancer development and is induced under hypoxic conditions [65]. HIF1α directly binds to the hypoxia response element (HRE) of the miR-210 promoter. Therefore, miR-210 plays an important role in regulating cellular adaption to hypoxia, suggesting that targeting miR-210 may be a novel approach for the prevention and/or treatment of cancer.

### 4.2. Association between OS, miRNA and Angiogenesis

Angiogenesis is the process of generating new blood vessels from preexisting vasculature and is required for many functions, such as tissue repair, organ regeneration, cancer development and metastasis [66]. The angiogenesis process is regulated by several cytokines and growth factors, such as vascular endothelial growth factor (VEGF), transforming growth factor β (TGFβ), angiopoietin 1 (Ang-1) and placental growth factor, platelet-derived growth factor (PDGF) β [67,68,69,70]. VEGF acts as an effector to control endothelial cell proliferation and new vessel formation. The HIF1α/ROS axis activates tissue-specific angiogenesis through the upregulation of VEGF and its receptors VEGFR1 and VEGFR2. By contrast, VEGF induces ROS production by promoting NADPH oxidase in endothelial cells. ROS can also modulate VEGFR activation, phosphorylation and polymerization. A report indicated that genotoxic stress-induced miR-494 expression suppressed DNA repair and angiogenesis through regulation of MRE11a/RAD50/NBN (MRN) complex in endothelial cells [71]. Moreover, VEGF signaling is regulated by MRN complex in vitro and in vivo. Alternatively, ROS stimulate the MAPK pathway and promote the expression of VEGF. A previous study demonstrated that oxidized phospholipids interact with VEGFR2 and induce angiogenesis through the Src signaling pathway [72]. Other mechanisms of ROS-mediated angiogenesis are the ataxia telangiectasia mutated gene (ATM)/p38α pathway and Sirtuin 1 (SIRT1). Previous studies have indicated that ATM functions in the cell cycle regulation, DNA damage repair and oxidative defense [73]. ATM promotes endothelial cell proliferation and facilitates angiogenesis [74]. Previously, the subtype of histone H2A, called H2AX, can be phosphorylated (γH2AX) and is involved in DNA damage response. Economopoulou et al. [75] group indicated that H2AX is required for endothelial cells to sustain their growth under hypoxia and is important for hypoxia-driven neovascularization. Wilson et al. [76] have shown that miR-103 suppresses developmental and pathological angiogenesis through inhibition of three prime exonucleases 1 in endothelial cells. On the other hand, Yang and co-workers demonstrated that overexpression of miR-328-3p suppressed cell proliferation and promoted radiosensitivity of osteosarcoma cells through suppression of H2AX in vitro and in vivo [77]. Recently, Marampon et al. group demonstrated that NRF2/antioxidant enzymes/H2AX/miRNAs (miR-22, miR-34a, miR-126, miR-146a, miR-210 and miR-375) axis act as potential candidates in radiosensitizing therapeutic strategy for rhabdomyosarcoma clinical treatment [78]. SIRT1, also known as NAD-dependent deacetylase sirtuin-1, has been demonstrated to regulate cellular functions including oxidative stress, apoptosis and aging via deacetylation of a variety of substrates [79]. A report indicates that inhibition of SIRT1 with either an inhibitor or siRNA leads to increased ROS levels, suggesting an association between SIRT1 and ROS. MiR-138, miR-181 and miR-199 have been shown to directly target and inhibit SIRT1 expression in various cell lines [80,81,82]. MiR-181 is induced by treatment with a high-fat diet and results in repressed SIRT1 expression and insulin sensitivity in the liver [81]. In addition, HIF1α and SIRT1 are upregulated in miR-199a-depleted cells during normoxic conditions [83]. Moreover, SIRT1 is actually a direct target gene of miR-199a and is responsible for suppressing prolyl hydroxylase 2.

### 4.3. Association between OS, miRNA and Metastasis

Metastasis is a complicated process that includes invasion, intravasation into blood, extravasation to distant organs and growth [84]. Due to these multiple steps, few metastasizing tumor cells can survive and form micrometastases. A typical phenotype that leads to metastasis is EMT, which is a biological event by which epithelial cells undergo alterations that induce the development of a more aggressive mesenchymal phenotype [84]. Increasing evidence suggests that cancer cells during the metastasis process are killed by OS [85,86]. In addition, cancer cells are more sensitive to ROS than normal cells. Reducing ROS levels by treating with antioxidant inhibits tumor promotion of tumor progression in mouse models. ROS-mediated EMT regulation through TGFβ/Smad, E-cadherin, Snail, integrin, β-catenin, matrix metalloproteinases (MMPs) and miRNA has been documented [87,88,89]. Among these interactions, activation of TGFβ induces ROS production and leads to the promotion of SMAD and ERK1/2 phosphorylation. Moreover, the ROS/TGFβ axis regulates EMT through the interaction of NFκB, HIF1α and cyclooxygenase-2 (COX-2). A previous study indicated that MMP-3, MMP-10 and MMP-13 were directly upregulated by oxidative treatment and promoted cell invasion ability in NMuMG cells [90]. In addition, the activity of MMP-2 and MMP-9 were posttranscriptionally regulated by oxidant treatment [91,92]. These studies suggest that MMP expression or activity is modulated by OS, which is related to chronic inflammation, malignant transformation and the invasive potential of cells. Yoon et al. [93] demonstrated that sustained treatment with H_2_O_2_ enhances MMP2 activity via the PDGF, VEGF, phosphatidylinositol 3-kinase and NF-κB pathways in HT1080 cell lines. Song and coworkers reported that the expression of miR-509 is significantly more downregulated in breast cancer than it is in normal tissues [94]. Overexpressed miR-509 abrogated cell growth, migration and invasion through inhibition of the target gene SOD2, which is a crucial effector in the production of ROS.

### 4.4. Association between OS, miRNA and Metabolism

Tumor progression is characterized by the occurrence of metabolic alterations, including those in glycolysis, fatty acid oxidation (FAO) and oxidative phosphorylation [95,96]. The connection and reciprocal regulation between the metabolism and the redox balance of tumor cells have been shown. For this reason, it is important to determine the major metabolic pathways that are the main controllers of the ROS homeostasis of cancer cells. Glucose is converted to glucose-6-phosphate by hexokinase enzyme and triggers a series of downstream enzyme-catalyzed reactions. It is an essential pathway for providing nutrients, metabolites and energy to cells. In 1924, Otto Warburg proposed a theory suggesting that tumor cells tend to exhibit glycolysis regardless of the presence of oxygen [97]. Accumulating evidence has shown that metabolites produced by glucose metabolism are major regulators of the redox homeostasis of tumor cells [98]. Cancer cells demonstrate increased sensitivity to glucose-deprivation-induced cytotoxicity compared with that in normal cells by restricting the burden of ROS. Moreover, inhibition of lactate dehydrogenase-A by a specific inhibitor, FX11, reduced intracellular ATP and promoted OS, which suppressed tumor progression in lymphoma and pancreatic cancer. Sala et al. [10] have shown that miR-21 is upregulated by glucose treatment and inhibits ROS homeostatic genes such as NRF2, SOD2 and KRIT1. Furthermore, other metabolic enzymes, such as TIGAR and ALDH4, decrease ROS production by either inhibiting glycolysis and inducing NAPDH production or enhancing mitochondrial function [99,100]. FAO consists of multiple processes by which fatty acids are broken down by cells to produce ATP and generate biosynthetic pathways. In general, the β-oxidation reaction takes place in mitochondria. FAO causes ROS formation and contributes to the enhanced development of nonalcoholic fatty liver disease (NAFLD) [101]. In hypoxia, HIF-1 suppression of medium-chain acyl-CoA dehydrogenase (MCAD) and light chain acyl-CoA dehydrogenase (LCAD) expression inhibits FAO and ROS production while promoting cell growth of liver cancer cells [102]. The expression levels of LCAD in HCC specimens were analyzed and found to be negatively correlated with survival. These findings indicate the relevance of FAO suppression in the progression of cancer. Previous studies have identified miR-33a/b as an intronic miRNA located with the sterol regulatory element binding factor (SREBP) 1 and 2 genes [103]. These two miRNAs cotranscribe with their host gene and regulate high density lipoprotein (HDL) biosynthesis.

### 4.5. Association between OS, miRNA and Cancer Stem Cells

Cancer cells are believed to be derived from a small subset of tumor cells that have a high capacity for self-renewal and differentiation—namely, cancer stem cells (CSCs) or tumor-initiating cells [104]. Increasing evidence indicates that miRNAs function as regulators of CSCs and are associated with ROS production during tumor progression and cancer development. Some miRNAs, such as let-7a, miR-21, miR-34a, miR-200 and miR-210, are potentially involved in the modulation of ROS production in CSCs [105,106,107,108,109]. A previous study showed that let-7 acts as a negative regulator of CSC-mediated function by targeting PTEN and LIN28b in prostate and pancreatic cancer. Recently, OS reduced let-7 expression in a p53-dependent manner in various cancer cells. Some experimental studies revealed that the expression of miR-21 is remarkably increased in CSC subpopulations compared to the expression in the hypobromite non-CSC counterparts in vitro and in vivo. Notably, knocking down miR-21 suppressed cell migration, invasion and EMT phenotype in breast cancer CSCs. Moreover, OS induced miR-21 expression and promoted cell migration and self-renewal in prostate and pancreatic CSCs. Another report indicates that miR-21 enhances ROS production via the MAPK pathway and suppresses SOD2, SOD3 and sprouty homolog 2 (SPRY-2) expression [110]. Additionally, a number of studies have revealed that miR-34a suppresses CSC-related genes, such as CD44, and EMT makers and subsequently attenuates cell invasion, metastasis and self-renewal capacity [111]. The interplay between ROS and miR-34 has been documented. The expression of miR-34 is induced by OS in stromal and tumor cells. The first evidence miR-200 was associated with stem cell phenotype, reported in 2009 [112]. Moreover, all five members of the miR-200 family were downregulated in human breast CSCs as well as in normal human and murine mammary stem/progenitor cells [112]. Mechanistically, miR-200 suppresses the expression of B lymphoma Mo-MLV insertion region 1 homolog (Bmil-1), Suz12, and Notch homolog 1 (Notch1), which are known regulators of CSC and EMT phenotypes, and inhibits the CSC self-renewal capacity. MiR-210 expression is enriched in MCF-7 spheroid cells and CD44^+^/CD24^−^ MCF7 cells compared with MCF-7 parental cells [113]. Overexpression of miR-210 enhances proliferation, self-renewal capacity, migration and invasion through inhibition of E-cadherin in vitro and in vivo. Thus, these observations indicate that the miRNA/ROS axis plays important roles in multiple events related to CSCs.

### 4.6. Association between OS, miRNA and Senescence

Cellular senescence is characterized by the expression of senescence-associated β-galactosidase (SA-β-gal), overexpression of the cyclin-dependent kinase (CDK) inhibitor, senescence-associated secretory phenotype (SASP), telomere shortening and persistent DNA damage response (DDR) [114]. ROS cause cell senescence by stimulating the DDR pathway to stabilize p53 and promote CDK inhibitor gene expression. In fact, p53 acts as a master regulator in the cellular response to OS. Mechanically, p53 can decrease ROS levels and repair DNA damage in cells. In contrast, it can also enhance ROS production and promote cell apoptosis or senescence [115]. Several reports indicate that p53 reduces intracellular ROS levels by promoting antioxidant reactions. Several miRNAs, including miR-21, miR-22, miR-29, miR-34a, miR-106b, miR-125b, miR-126, miR-146a, the miR-17-92 cluster, the miR-200 family and miR-210, have been identified to be differentially expressed in senescent cells and to be involved in cellular senescence [116,117,118,119,120,121,122]. Notably, miR-34a was found to promote cellular senescence by inhibiting SIRT1 expression in a variety of tissues. Another group indicated that miR-34a and miR-335 promote premature cellular senescence by targeting antioxidative enzymes. Furthermore, miR-217 induces a premature senescence-like phenotype and represses angiogenesis by inhibiting the expression of target gene SIRT1 in endothelial cells [123]. In addition, miR-92a was found to exacerbate endothelial dysfunction under OS exposure by directly targeting SIRT1, Krüppel-like factor 2 (KLF2) and KLF4 genes [124]. Additionally, Liu et al. group demonstrated that knockdown of miR-92a promoted cell growth, decreased caspase 3 activity and ROS through regulation of NRF2-KEAP1/ARE signal pathway [125].

## 5. ROS-Mediated Therapeutic Strategies in Cancer

OS clearly plays a role in the development of cancer, metastasis and chemotherapeutic resistance. In a strategy to modulate ROS-mediated effects, these biochemical characteristics of tumors are directly impaired. In light of recent studies, the strategy of inhibiting metabolic pathways, targeting NADPH oxidase and ROS scavenging mechanisms represent promising therapeutic options for treatments [126]. The other strategy is to target tumor cells with oxidation-promoting agents that either enhance ROS production or inhibit cellular antioxidants. NADPH oxidase plays an important role in regulating ROS production. Several inhibitors have been demonstrated to reduce NADPH function. In general, diphenylene iodonium (DPI) and apocynin are NADPH inhibitors [127,128,129]. DPI can inhibit XOD and the proteins of mitochondrial ETC and block flavoprotein. In another strategy, ROS scavenging enzymes are enhanced and used for anticancer therapy [130]. GSH, GST, SOD, GPX, and catalase are able to suppress tumor formation. There are several analogs of GSH drugs, such as *N*-acetylcysteine (NAC), YM737 and Telcyta, used for cancer treatment [131]. NOV-002, an agent containing oxidized GSH, improved the efficacy of cyclophosphamide to treat colon cancer by controlling the ratio of GSH to GSSG and promoting S-glutathionylation [131]. Yang and coworkers indicated that lithocholic acid treatment and bile duct ligation model promoted c-Myc/miR-27/prohibitin 1 axis, with the consequence of repressing NRF2 expression and ARE binding, resulting in decreased suppressed GSH synthesis and antioxidant ability in chronic cholestatic liver injury [132]. Another study reported that the rate-limiting GSH biosynthetic heterodimeric enzyme γ-glutamyl-cysteine ligase (GCL) was regulated by miR-433 [133]. Ectopic of miR-433 in HUVEC inhibited GCL expression in an NRF2-independent manner. Moreover, inhibition of miR-433 prevented TGFβ-mediated GCL downregulation and fibrogenesis in hepatic cells. Recently, Cheng et al. [134] demonstrated that miR-30e expression was suppressed in an atherosclerosis (AS) model. MiR-30e regulates Snai1/TGF-β/Nox4 expression to modulate ROS. These findings provide novel insights on miRNAs in the anti-ROS pathway, in which miRNA-30e may represent a novel target for AS.

## 6. Conclusions

**Figure 3:**
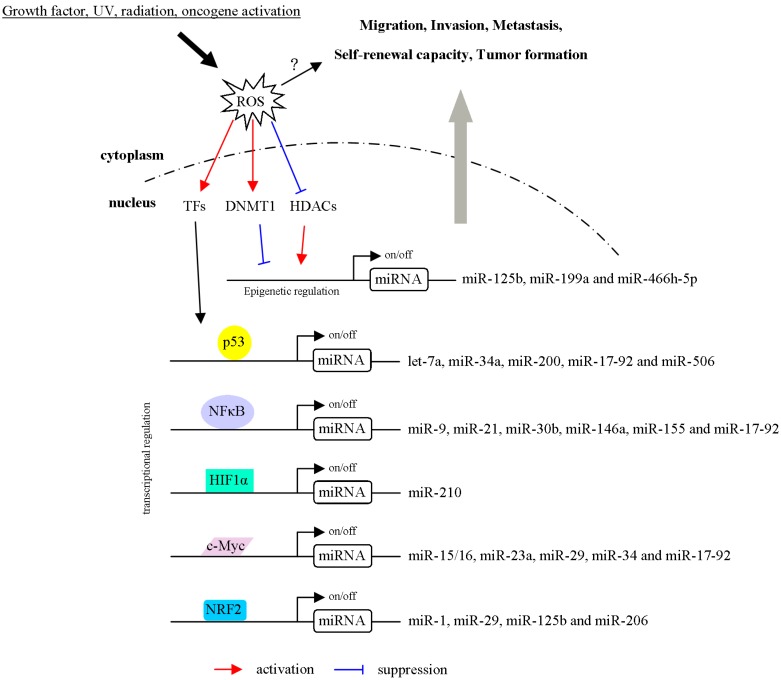
Schematic model showing mechanisms in which ROS regulates the biogenesis and transcription of miRNAs. ROS activate or inhibit epigenetic, transcriptional regulations of miRNA expression. For example, miRNAs are regulated by ROS through modulation of chromatin remodeling factors (DNMT1 and HDACs). In addition, ROS induces or represses transcriptional factor (p53, NFκB, HIF1α, c-Myc and NRF2) to regulate miRNA expressions. Furthermore, ROS/TF/miRNA axis controls cell migration, invasion, metastasis, self-renewal capacity and tumor formation.

Overall, many studies have been conducted to elucidate the molecular mechanisms underlying the ROS/miRNA axis and its role in tumorigenesis (Figure 3). Moreover, miRNAs networks that modulate OS in cancer are comprehensively listed in Table 1. Indeed, ROS and miRNAs exhibit overlapping characteristics in tumorigenesis. ROS, as upstream regulators, modulate miRNA expression through transcriptional, posttranscriptional and epigenetic regulation, respectively. On the other hand, miRNAs disrupt ROS production (downstream mediator) and are involved in ROS-mediated functions. MiRNAs and ROS can act either synergistically or antagonistically to regulate cancer progression. However, many details of their interaction remain unclear and need to be further investigated. MiRNAs/ROS-mediated phenotypes depend on the net result of the downstream molecules and multiple signaling pathways in the specific context. There are still many limitations to treatment because ROS play dual roles in cancer progression. As discussed in this review, the functional roles of miRNA in cellular adaptation to ROS are different in cells based on tissue and cell-type specific effects. These observations raise the possibilities to apply specific miRNAs as therapeutic targets in different contexts. Advantages of using miRNA-target therapy include the conservation of miRNA across multiple species with known sequences and the ability to target multiple genes within defined pathways. Notably, several miRNA-based therapies are being developed. For example, the locked nucleic acid (LNA)-modified anti-miR-122 is the first miRNA-targeted therapy to treat HCV in clinical trials. The association between ROS-mediated function and miRNA regulation provides opportunities for developing novel anticancer strategies.

## Figures and Tables

**Table 1:** ROS-related miRNAs and their potential mechanisms in cancers.

miRNA	Regulation Mechanism ^a^	ROS Production ^b^	Expression in Cancer ^c^	Cell/Cancer Types	Molecules, Cellular Processes and Signaling Pathways Involved ^d^	References
Let-7a	OS, p53	✓	Down	CSC, prostate cancer, pancreatic cancer	PTEN, LIN28b	[105,135]
miR-1	NRF2, HDAC4	✓	Down	Non-small cell lung cancer	NRF2, KEAP1, glucose metabolism, tumor growth	[49]
miR-15/16	c-Myc	✓	Down	Skin, colon cancer	FGF2, HIF-2α, senescence-like phenotype, angiogenesis, metastasis	[30]
miR-21	Glucose, NFκB, STAT3	✓	Up	CSCs, lung cancer, liver cancer, colorectal cancer	MAPK pathway, cell migration, invasion and EMT phenotype, self-renewal ability	[41,110]
miR-23a	c-Myc	✓	-	Cardiac disease, myeloma	Glutaminase, MnSOD, apoptosis, cell growth	[136,137,138]
miR-29	c-Myc, H_2_O_2_, NRF2	✓	Dual role	Ovarian cancer, lung cancer, lymphoma	SIRT1, senescence, proliferation, apoptosis	[50,139,140,141,142]
miR-33a/b	-	✓	Down	Liver	HDL biosynthesis, apoptosis, OS resistance	[103]
miR-34	OS, c-Myc, p53	✓	Down	Stromal cells, CSC, bladder cancer, lung cancer	CD44, EMT markers, SIRT1, senescence, metastasis	[33,111,143]
miR-17-92	c-Myc, p53, NFκB	✓	Up	Lung cancer,	Vitamin D, Senescence, apoptosis	[120,144,145,146]
miR-92a	-	✓	Up	Endothelial cells	SIRT1, KLF2, KLF4	[124,125]
miR-125b	DNMT1, H_2_O_2_, NRF2	✓	Dual role	Ovarian cancer, liver	Epigenetic regulation	[51,57]
miR-181	-	✓	Up	Macrophagy, HCC	SIRT1, insulin sensitivity, NFκB activity, apoptosis	[80]
miR-199a	DNMT1, H_2_O_2_	✓	Down, (hypermethylation)	Ovarian cancer	HIF1α, SIRT1, Epigenetic regulation	[57,83]
miR-200	P53, H_2_O_2_	✓	Down	CSC, breast cancer, liver cancer	Bmil-1, Suz12, Notch-1, self-renewal capacity, EMT markers, senescence	[34,35]
miR-210	Hypoxia	✓	Up	CSCs	E-cadherin, Hypoxia, proliferation, self-renewal capacity, migration and invasion, senescence	[65,109] [113]
miR-217	-	-	Dual role	Endothelial cells	SIRT1, Angiogenesis, premature senescence-like phenotype	[123]
miR-466h-5p	ROS, HDAC2	-	-	Mouse ovarian epithelial	BCL2L2, apoptosis	[58]
MiR-506	P53	✓	Down	Lung cancer	NFκB signaling pathway	[44]
miR-509	-	✓	Down	Breast cancer	SOD2, Cell growth, migration and invasion	[94]

^a^: MiRNAs are regulated by upstream transcriptional factor, ROS or hypoxia, as indicated. -: Information is unavailable. ^b^: ✓: MiRNAs are responsible for producing ROS. -: Information is unavailable. ^c^: Expression level of miRNAs in cancer. Up: upregulated in cancer, Down: downregulated in cancer, Dual role: up- or downregulated in cancer. ^d^: Downstream molecules, signaling pathways and phenotypes involved in miRNA-mediated functions.
